# Study of the Predictive Validity of the Burnout Granada Questionnaire in Police Officers

**DOI:** 10.3390/ijerph17176112

**Published:** 2020-08-22

**Authors:** Emilia I. De La Fuente-Solana, Elena Ortega-Campos, Keyla Vargas-Roman, Gustavo R. Cañadas-De la Fuente, Tania Ariza C., Raimundo Aguayo-Extremera, Luis Albendín-García

**Affiliations:** 1 Faculty of Psychology, Campus de la Cartuja s/n, University of Granada, 18011 Granada, Spain; edfuente@ugr.es (E.I.D.L.F.-S.); keyvarom@ugr.es (K.V.-R.); 2 Department of Psychology, Faculty of Psychology, University of Almería, 04120 Almería, Spain; 3 Faculty of Education, Campus de la Cartuja s/n, University of Granada, 18011 Granada, Spain; grcanadas@ugr.es; 4 Faculty of Education, Avda de la Paz, 137, Universidad Internacional de La Rioja, 26006 Logroño, La Rioja, Spain; tania.ariza.castilla@unir.net; 5 Faculty of Psychology, Campus de Somosagua s/n, Complutense University of Madrid, 28223 Madrid, Spain; raaguayo@ucm.es; 6 Andalusian Health Service, Avenida del Sur, 11, 18014 Granada, Spain; lualbgar1979@ugr.es

**Keywords:** burnout, GBQ, MBI, police officers, validity

## Abstract

Professionals with burnout have negative physical and psychological effects, with adverse consequences in their workplace. Burnout mainly affects assisting professions; amongst them, police work is one of the professions at risk of suffering from this syndrome. The aim of this research is to study the adequacy of the Maslach Burnout Inventory and Granada Burnout Questionnaire instruments to measure burnout in police officers through the study of the reliability and validity (concurrent and predictive) of these instruments. A cross-sectional study was carried out. The sample was composed of 1884 police officers, mostly men (85.4%), with an average age of 35.04 (SD = 8.30). The Maslach Burnout Inventory and Granada Burnout Questionnaire were used to measure burnout. The results obtained in this study support the adequacy of both instruments for measuring burnout. The correlation coefficients between the dimensions are significant, with a medium-high magnitude. Participants with burnout had significantly higher scores in emotional exhaustion and depersonalization and lower scores in personal accomplishment in both instruments. The area under the curve estimated for the Granada Burnout Questionnaire provided evidence of the predictive validity of the instrument. The police profession needs validated and sensitive tools to identify police changes in the dimensions of burnout. The Granada Burnout Questionnaire instrument correctly classifies burnout in police professionals.

## 1. Introduction

Burnout is a psychological syndrome characterized by physical, emotional and mental exhaustion, which occurs as a result of chronic exposure to stressors [1]. Workers with burnout experience emotional exhaustion (EE), or feelings of physical overexertion and emotional exhaustion as a result of interactions with service users; depersonalization (D), or cynical attitudes and responses towards service users; and a feeling of low personal accomplishment (PA), or loss of confidence and negative self-concept [2].

Moreover, burnout has negative effects, both physical and psychological, for the professionals who present it, as well as unfavorable consequences in their workplace (absenteeism, incapacity for work, impaired attention or increased number of errors at work) [3]. Given the importance of burnout syndrome in workers, it has recently been recognized as an occupational disease and is expected to be included in the International Classification of Diseases in 2022 (ICD-11) [4].

This syndrome mainly affects professions in which the job is to provide assistance (doctors, nurses, firefighters, police), but it has not been studied equally in all the professions it affects. In the field of health care, burnout is widely studied, both in empirical studies, as in systematic reviews, and meta-analyses [5,6,7]. The studies carried out indicate that prevention of burnout syndrome is important, with the aim of offering better patient care. On the contrary, in other professions with burnout syndrome, such as security forces, specifically the police, it is a poorly addressed issue [8].

Police work is one of the professions at risk of burnout, receiving high task demands (physical, psychological, social and work organization) that require permanent physical and psychological efforts from police officers [9,10]. Several studies indicate the difficulties experienced in police work include high job stress, lack of support and high incidence of mental health problems. They are exposed to a high level of work-related stress [11], which makes it essential to study their work well-being [12]. On the other hand, high levels of the syndrome are related to a greater occurrence of violent behavior, lack of control and deterioration of the correct performance of work. For an organization, the appearance of the syndrome not only affects its professionals, who can reach very high levels of suicidal ideation, but also leads to lower work efficiency and higher absenteeism [13].

The Maslach Burnout Inventory (MBI) [1] has been used almost unanimously as an instrument for assessing burnout syndrome, which suggests that most researchers support this approach. This questionnaire proposes the conceptualization of the syndrome that we are using, and many researchers defend its convergent validity, discriminant, dimensionality and, in general, its good psychometric indicators for the evaluation of burnout syndrome. The Granada Burnout Questionnaire (GBQ) has been created as an alternative to the Spanish adaptation of the MBI [14]. The GBQ uses the same theoretical structure as the MBI and has good psychometric indicators, updated standards and the adequate reliability and validity to evaluate burnout syndrome in Spanish police [13]. In addition, its publication in open access makes it possible for any researcher to make use of it. As with any other psychological assessment tool, we consider it necessary to update and deepen the study of the psychometric properties of the GBQ.

The aim of this research is to study the adequacy of the MBI and GBQ instruments to measure burnout in police officers through the study of the reliability and validity (concurrent and predictive) of these instruments.

## 2. Materials and Methods

### 2.1. Sample and Setting

The sample of this work consisted of a total of 1884 police officers. They were mostly men (85.4%) with a mean age of 35.04 (SD = 8.30), and the age range varied between 20 and 64 years. The study was conducted on-site at police stations while the officers were on duty. Participation in the study was voluntary, individual and anonymous, and the time it took to complete the questionnaires was approximately 45 min.

### 2.2. Variables and Instruments

The following variables were collected: gender (male vs. female), age, marital status (single, married or divorced or separated), number of children, level of education (primary school, high school or university), work scale (basic, sub-inspector or executive), work shift (morning, afternoon, morning and afternoon shifts) and months of seniority at work.

The three dimensions of burnout have been measured with the Maslach Burnout Inventory (MBI) [1] in a version adapted to the Spanish population [14]. This questionnaire consists of 22 items with a seven-point Likert response. The MBI has three dimensions or scales: emotional exhaustion (9 items), depersonalization (5 items) and personal accomplishment (8 items).

The Granada Burnout Questionnaire (GBQ) is also used to measure burnout. The GBQ [13] is designed to measure burnout, following the guidelines proposed by Downing (2006) [15]. It was created based on the operational definition of burnout, proposed by Maslach, Schaufeli and Leiter (2001) [16], to define the dimensions: emotional exhaustion, depersonalization and personal accomplishment. The GBQ consists of a total of 26 items, with a Likert response format of 5 alternatives (1 = totally disagree and 5 = totally agree) grouped in 3 dimensions: emotional exhaustion (9 items), depersonalization (7 items) and personal accomplishment (10 items). The items measure the dimensions of burnout positively or negatively, so 11 items must be reversed when performing the revision (items 1, 4, 10, 12, 13, 19, 20, 23, 24, 25 and 26).

### 2.3. Design and Procedure

A cross-sectional study was carried out. The National Committee for Workplace Risks of the Joint Trade Union of the Police (Unified Police Union; UPU) was contacted. The participants were reached with the collaboration of this committee, which helped to coordinate the accession of the questionnaires.

### 2.4. Ethics

This study was approved by the Ethics Committee of the University of Granada (393/CEIH2017) and was conducted in accordance with the ethical standards of the Declaration of Helsinki (2008). All data in the study were processed in accordance with Spanish data protection legislation [17].

### 2.5. Data Analysis

Cronbach’s alpha reliability coefficient has been calculated for the emotional exhaustion, depersonalization and personal accomplishment dimensions of the MBI and GBQ instruments. For evaluation of the reliability coefficients, the recommendations of George and Mallery (2003) [18] have been followed. Frequencies and percentages have been estimated for qualitative variables, descriptive variables (mean, standard deviation, minimum and maximum) and Pearson’s correlation coefficient for quantitative variables. To check for differences between groups, the Student’s t-test for independent groups, the effect size index (Cohen’s d) and their confidence interval have been estimated. The effect size index reports the real importance of the results obtained in the significance tests [19,20]. The Golembiewski, Munzerider and Stevenson (1986) [21] model was used to classify participants into highs and lows in the dimensions of burnout, in reference to their MBI scores. As a complement to the study of GBQ validity tests, the area under the curve (AUC) and its CI95% have been estimated for each dimension of burnout, indicating the predictive validity, which can vary from 0 (perfect negative prediction) to 1 (perfect positive prediction). An AUC of 0.50 corresponds to a prediction equal to random. An AUC between 0.56 and 0.64 is considered a small effect, above 0.64 is an average effect, and an AUC greater than 0.71 is a large effect [22].

## 3. Results

### 3.1. Sociodemographic Sample Variables

According to the marital status of the participants, 48.2% were single and 47.9% were married. Almost half of the sample had no children (49.1%), 22.2% had one child and 22.8% had two children. Attending to the level of education, 59.7% had a high school education and 32.2% had a university education. In the work variables, 89% of the participants had their position as basic scale, 7% as sub-inspector and 4% as executive scale. Almost half of the participants (48.6%) worked shift work, 23.9% worked morning and afternoon hours, 14.5% worked in the morning and 9.1% worked in the afternoon. In terms of seniority at the workplace, the average number of months worked was 93.17 (SD = 109.07) (Table 1).

### 3.2. Reliability of the MBI and GBQ

Reliability has been calculated for the dimensions of the MBI: emotional exhaustion (α = 0.894, CI 95% (0.887, 0.901)), depersonalization (α = 0.688, CI 95% (0.665, 0.710)) and personal accomplishment (α = 0.846, CI 95% (0.836, 0.857)); and for the GBQ dimensions: emotional exhaustion (α = 0.869, CI95%(0.859, 0.877)), depersonalization (α = 0.849, CI 95% (0.838, 0.859)), and personal accomplishment (α = 0.799, CI 95% (0.785, 0.812)).

### 3.3. Descriptive of the MBI and GBQ

Mean scores, standard deviation and minimum and maximum values have been estimated for the subscale’s emotional exhaustion, depersonalization and personal accomplishment of the MBI and GBQ (Table 2). In the MBI, on the emotional exhaustion scale, participants had an average score of 12.82 (SD = 10.77), with a score range between 0 and 54. On the depersonalization scale, they got an average score of 7.55 (SD = 5.97), with a range of values between 0 and 30. On the personal accomplishment scale, they had an average score of 35.46 (SD = 9.68), with values between 0 and 48. In the GBQ, on the emotional exhaustion scale, they had an average score of 18.45 (SD = 7.50), with values between 9 and 45. The depersonalization scale had an average score of 12.75 (SD = 4.79) and values between 7 and 35. Finally, on the personal accomplishment scale, they got an average score of 40.26 (SD = 8.01), with a range of values between 10 and 50.

Pearson’s correlation coefficients between the MBI and GBQ scores have been estimated (Table 3). All coefficients are statistically significant. The emotional exhaustion scales of both instruments obtained an r = 0.706. The depersonalization scales of the instruments have an estimated value of r = 0.331. Finally, between the scales of personal accomplishment, a value of r = 0.479 was obtained.

### 3.4. Differences in MBI and GBQ according to Burnout

In order to verify whether the MBI and CBG correctly discriminate between the yes and no burnout groups, the mean scores, standard deviations, t-Student, *p*-value, Cohen’s d and IC 95% of the effect size have been estimated for the MBI and CBG subscales, according to the level of burnout of the participants. For the classification of participants with burnout, the recommendations of Golembiewski and Munzerider (1988) [23] and Golembiewski, Munzerider and Stevenson (1986) [21] have been followed. In the MBI, statistically significant differences have been found in the three dimensions of MBI between participants with and without burnout. The effect size index has been estimated for the comparisons made, obtaining for the emotional exhaustion dimension a Cohen d value equal to 2.39 CI 95% (2.23, 2.55), for the depersonalization dimension d = 1.86 CI 95% (1.70, 2.01) and for the personal accomplishment dimension d = 0.94 CI 95% (0.79, 1.08).

In the GBQ, statistically significant differences have been found in the three dimensions of GBQ between the burnout group and the non-burnout group. The estimated effect size index takes values of 1.54 CI 95% (1.38, 1.68) in emotional exhaustion, 0.94 CI 95% (0.79, 1.08) in depersonalization and 1.35 CI 95% (1.21, 1.50) in personal accomplishment (Table 4).

### 3.5. AUCs for MBI and GBQ

The area under the curve (AUC) and its 95% confidence interval have been estimated for each of the MBI and GBQ dimensions. Table 5 shows the values obtained. In the MBI, an AUC = 0.948 CI 95% (0.939, 0.958) was obtained for the emotional exhaustion dimension, AUC = 0.886 CI 95% (0.858, 0.914) for the depersonalization dimension and AUC = 0.251 CI 95% (0.213, 0.288) for the personal accomplishment dimension. In the CBG, an AUC = 0.854 CI 95% (0.830, 0.877) was estimated for the emotional exhaustion dimension, AUC = 0.729 CI 95% (0.691, 0.767) for the depersonalization dimension and AUC = 0.187 CI 95% (0.159, 0.216) for the personal accomplishment dimension.

## 4. Discussion

Police work is related to a high level of stress, both by the type of tasks they do and the working conditions in which they perform [10,11], something that also happens in other security groups [24]. Maintaining stressful situations over time can lead to the development of burnout by police officers [25] and cause deterioration in the performance of their functions (quality of work, absenteeism and abandonment of work) [26]. The relationship between burnout, stress and the health of police officers needs special attention from investigators. The presence of stress and burnout can mask physical health problems in police officers (obesity, hypertension, heart problems and other diseases). In order to respond to this problem, it is essential to carry out longitudinal studies and implement programs to reduce stress and promote healthy living habits in police officers [27,28,29,30].

As has already been stated at the beginning of this work, burnout is a psychological syndrome that was defined nearly four decades ago [1]. Not only does it remain current, but the severity of its consequences on the people who develop it starts to be recognized. Hence its future inclusion in the International Classification of Diseases (ICD-11) of the World Health Organization (2019) [4]. All of the above makes it essential to have both preventive programs of the syndrome and instruments that identify workers who present burnout and the phase they are in [16,31,32].

The objective of this study was to verify the reliability and validity of the MBI and GBQ in the measurement of burnout and to provide evidence of the validity of the GBQ in a sample of police officers. To meet the objectives of this work, two instruments measuring burnout (MBI and GBQ) had been administered to a sample of police officers. The MBI has a wide tradition in its use, both in professional practice and in scientific research, being validated in different contexts and languages [33,34,35]. The results obtained in this study provide empirical evidence on the reliability and validity of the MBI and GBQ instruments in the Spanish population. The reliability indices calculated in this study indicate that both instruments present adequate internal consistency [18]. The reliability indices of the GBQ and MBI present similar values, which supports the hypothesis that the GBQ can be an alternative to the Spanish version of the MBI.

In this work, we have found significant correlation coefficients with medium-high magnitudes between the dimensions of burnout between both instruments. These data support the hypothesis that both instruments measure the burnout construct formed by the dimensions of emotional exhaustion, depersonalization and personal accomplishment and support the fact that GBQ can be used as an instrument to measure burnout [36].

Taking as a reference the differences between the groups with and without burnout [21,23], it was found that the participants of the group with burnout had higher scores in EE and D and lower scores in PA, both in MBI and GBQ. The effect size index estimated in the three dimensions of the GBQ indicates large effect values. These data support the excellent performance of the GBQ and its ability to discriminate between workers with and without burnout.

The AUCs estimated for GBQ dimensions provide evidence of the predictive validity of the instrument. The values found indicate the proper functioning of the instrument. Following Rice and Harris (2005) [22], AUCs higher than 0.71 indicate great effects. The results are in line with outcomes found in other studies regarding the estimation of the predictive validity of instruments of burnout [37,38,39].

The police profession is associated with a high degree of commitment and a risk of burnout [40,41], so it is essential to have tools that are validated for the target population and sensitive in order to identify changes in the dimensions of burnout in the police. The GBQ instrument presents, taking as a criterion the MBI, a good performance in the measurement and identification of police professionals who present burnout. The results presented in this study indicate that the GBQ has concurrent and predictive validity in measuring police burnout and has the ability to identify police officers with burnout.

In view of future research, it would be advisable to conduct longitudinal studies with the objective of studying the behavior of burnout syndrome over time, although these types of studies are complicated to perform in groups difficult to access, such as police officers [42].

## 5. Conclusions

This work presents data on the proper functioning of GBQ to measure and identify burnout syndrome in a sample of police officers. The performance of the GBQ instrument has been compared with the MBI, an instrument widely used internationally to measure burnout. This paper presents evidence of the good functioning of GBQ and its predictive capacity for burnout.

## Figures and Tables

**Table 1:** Sociodemographic and labor variables.

	%(Frequency)		%(Frequency)
**Gender**		**Work Scale**	
Male	85.4(1604)	Basic	89(1676)
Female	14.6(274)	Sub-inspector	7(132)
**Marital status**		Executive	4(76)
Single	48.2(884)	**Work Shift**	
Married	47.9(878)	Morning	14.5(260)
Separated/Divorced	3.8(69)	Afternoon	9.1(164)
**Number of children**		Morning and afternoon	23.9(430)
0	49.1(789)	Shifts	48.6(874)
1	22.2(356)	**Education**	
2	22.8(367)	Primary School	8.2(150)
3+	6(95)	High School	59.7(1097)
		University	32.2(591)

N = 1884.

**Table 2:** Mean, SD, minimum and maximum of the MBI and GBQ subscales.

	Mean	SD	Minimum	Maximum
MBI Emotional Exhaustion	12.82	10.77	0	54
MBI Depersonalization	7.55	5.97	0	30
MBI Personal Accomplishment	35.46	9.68	0	48
GBQ Emotional Exhaustion	18.45	7.50	9	45
GBQ Depersonalization	12.75	4.79	7	35
GBQ Personal Accomplishment	40.26	8.01	10	50

N = 1884; SD = standard deviation; MBI = Maslach Burnout Inventory; GBQ = Granada Burnout Questionnaire.

**Table 3:** Pearson correlation coefficients between MBI and GBQ scales.

	MBI Emotional Exhaustion	MBI Depersonalization	MBI Personal Accomplishment
**GBQ Emotional Exhaustion**	0.706 *	0.460 *	−0.333 *
**GBQ Depersonalization**	0.384 *	0.331 *	−0.504 *
**GBQ Personal Accomplishment**	−0.634 *	−0.420 *	0.479 *

N = 1884; MBI = Maslach Burnout Inventory; GBQ = Granada Burnout Questionnaire; * *p* < 0.001.

**Table 4:** Differences in scores on the MBI and BGQ instruments between yes and no burnout groups and effect sizes with IC95%.

	Burnout	M(SD)	t	*p*	d Cohen	IC 95%
MBI Emotional Exhaustion	No	10.45(8.33)	−28.44	<0.001	2.39	[2.23, 2.55]
	Yes	30.92(10.18)				
MBI Depersonalization	No	6.45(5.01)	−25.76	<0.001	1.86	[1.70, 2.01]
	Yes	15.98(5.99)				
MBI Personal Accomplishment	No	36.47(9.12)	13.06	<0.001	0.94	[0.79, 1.08]
	Yes	27.75(10.33)				
GBQ Emotional Exhaustion	No	17.25(6.58)	−18.82	<0.001	1.54	[1.38, 1.68]
	Yes	27.60(7.76)				
GBQ Depersonalization	No	12.25(4.36)	−10.16	<0.001	0.94	[0.79, 1.08]
	Yes	16.58(6.08)				
GBQ Personal Accomplishment	No	41.41(7.09)	15.53	<0.001	1.35	[1.21, 1.50]
	Yes	31.45(9.12)				

N = 1884; No Burnout: N = 1666; Yes Burnout: N = 218; SD = standard deviation; *p* = *p*-valor; IC = interval of confidence; MBI = Maslach Burnout Inventory; GBQ = Granada Burnout Questionnaire.

**Table 5:** Area under the curves for MBI and GBQ dimensions.

	AUC	SE	CI 95%
MBI Emotional Exhaustion	0.948 *	0.005	[0.939, 0.958]
MBI Depersonalization	0.886 *	0.014	[0.858, 0.914]
MBI Personal Accomplishment	0.251 *	0.019	[0.213, 0.288]
GBQ Emotional Exhaustion	0.854 *	0.012	[0.830, 0.877]
GBQ Depersonalization	0.729 *	0.019	[0.691, 0.767]
GBQ Personal Accomplishment	0.187 *	0.015	[0.159, 0.216]

N = 1884; No burnout: N = 1666; Yes Burnout: N = 218; AUC = area under the curve; SE = standard error; CI = confidence interval; MBI = Maslach Burnout Inventory; GBQ = Granada Burnout Questionnaire; * *p* < 0.001.

## References

[1] Maslach C., Jackson S.E. (1981). The Measurement of Experienced Burn-out. J. Organ. Behav..

[2] Maslach C., Leiter M. (2008). Early Predictors of Job Burnout and Engagement. J. Appl. Psychol..

[3] Davey D.M., Cummings G., Newburn-Cook C.V., Lo E.A. (2009). Predictors of Nurse Absenteeism in Hospital: A systematic review. J. Nurs. Manag..

[4] World Health Organization (2019). The Organization of Work and Stress. https://www.who.int/occupational_health/publications/stress/es/.

[5] Cañadas-De la Fuente G.A., Vargas C., San Luis C., García I., Cañadas G.R., De la Fuente E.I. (2015). Risk factors and prevalence of burnout syndrome in the nursing profession. Int. J. Nurs. Stud..

[6] Ortega-Campos E., Vargas-Román K., Velando-Soriano A., Suleiman-Martos N., Cañadas-de la Fuente G., Albendín-García L., Gómez-Urquiza J. (2020). Compassion fatigue, compassion satisfaction, and burnout in oncology nurses: A systematic review and meta-analysis. Sustainability.

[7] Schaufeli W.B., Leiter M.P., Maslach C. (2009). Burnout: 35 years of research and practice. Career Dev. Int..

[8] Turgoose D., Glover N., Barker C., Maddox L. (2017). Empathy, compassion fatigue, and burnout in police officers working with rape victims. Traumatology.

[9] Bakker A.B., Demerouti E. (2007). The job demands-resources model: State of the art. J. Manag. Psychol..

[10] Vuorensyrjä M., Mälkiä M. (2011). Nonlinearity of the effects of police stressors on police officer burnout. Policing.

[11] McCreary D.R., Fong I., Groll D.L. (2017). Measuring policing stress meaningfully: Establishing norms and cut-off values for the operational and organizational police stress questionnaires. Police Pract. Res..

[12] Basinska B.A., Dåderman A.M. (2019). Work values of police officers and their relationship with job burnout and work engagement. Front. Psychol..

[13] De la Fuente E.I., Lozano L.M., García-Cueto E., San Luis C., Vargas C., Cañadas G.R., Cañadas-De la Fuente G.A., Hambleton R.K. (2013). Development and validation of the granada burnout questionnaire in spanish police. Int. J. Clin. Health Psychol..

[14] Seisdedos N. (1997). MBI. Maslach Burnout Inventory.

[15] Downing S.M., Downing S.M., Haladyna T.M. (2006). Twelve steps for effective test development. Handbook of Test Development.

[16] Maslach C., Schaufeli W., Leiter M.P. (2001). Job burnout. Annu. Rev. Psychol..

[17] Organic Law 15/1999 of 13 December on the Protection of Personal Data. https://boe.es/buscar/act.php?id=BOE-A-19992-3750.

[18] George D., Mallery P. (2003). SPSS for Windows Step by Step: A Simple Guide and Reference. 11.0 Update.

[19] García J., Ortega E., De la Fuente L. (2011). The use of the effect size in JCR spanish journals of psychology: From theory to fact. Span. J. Psychol..

[20] Vacha-Haase T., Nilsson J.E., Reetz D.R., Lance T.S., Thompson B. (2000). Reporting practices and APA editorial policies regarding statistical significance and effect size. Theory Psychol..

[21] Golembiewski R.T., Munzerider R.F., Stevenson J.G. (1986). Stress in Organizations. Toward a Phase Model of Burnout.

[22] Rice M.E., Harris G.T. (2005). Comparing effect sizes in follow-up studies: ROC area, Cohen’s d, and r. Law Hum. Behav..

[23] Golembiewski R.T., Munzerider R.F. (1998). Phases of Burnout. Developments in Concepts and Applications.

[24] Schult T.M., Mohr D.C., Osatuke K. (2018). Examining burnout profiles in relation to health and well-being in the veterans health administration employee population. Stress Health.

[25] Rajaratnam S.M.W., Barger L.K., Lockley S.W., Shea S.A., Wang W., Landrigan C.P., O’Brien C.S., Qadri S., Sullivan J.P., Cade B.E. (2011). For the harvard work hours health and safety group. sleep disorders, health, and safety in police officers. JAMA.

[26] Noblet A.J., Rodwell J.J., Allisey A.F. (2009). Police stress: The role of the psychological contract and perception of fairness. Policing.

[27] Capitanelli I., Garbarino S., Magnavita N. (2017). Stress and cardiovascular risk in police. A systematic review. Eur. J. Public Health.

[28] Shiozaki M., Miyai N., Morioka I., Utsumi M., Hattori S., Koike H., Arita M., Miyashita K. (2017). Job stress and behavioral characteristics in relation to coronary heart disease risk among japanese police officers. Ind. Health.

[29] Vasileia V., Andrea F., Maria K., Sho S., Dahabreh I.J., Stefanos S.N. (2014). Law enforcement duties and sudden cardiac death among police officers in united states: Case distribution study. BMJ.

[30] Queirós C., Passos F., Bártolo A., Marques A.J., da Silva C.F., Pereira A. (2020). Burnout and stress measurement in police officers: Literature review and a study with the operational police stress questionnaire. Front Psychol..

[31] Kristensen T.S., Borritz M., Villadsen E., Christensen K.B. (2005). The copenhagen burnout inventory: A new tool for the assessment of burnout. Work Stress.

[32] Stamm B.H. The Concise ProQOL Manual 2010. http://proqol.org.

[33] Faye-Dumanget C., Carré J., Le Borgne M., Boudoukha P. (2017). French validation of the maslach burnout inventory-student survey (MBI-SS). J. Evaluation Clin. Pr..

[34] Kitaoka-Higashiguchi K., Ogino K., Masuda S. (2004). Validation of japanese research version of maslach burnout inventory-general survey. Shinrigaku Kenkyu.

[35] Loera B., Converso D., Viotti S. (2014). Evaluating the psychometric properties of the maslach burnout inventory-human services survey (MBI-HSS) among Italian Nurses: How many factors must a researcher consider?. PLoS ONE.

[36] De la Fuente E., García J., Cañadas G.A., San Luis C., Cañadas G.R., Aguayo R., de la Fuente L., Vargas C. (2015). Psychometric properties and scales of the granada burnout questionnaire applied to nurses. Int. J. Clin. Health Psychol..

[37] Knox M., Willard-Grace R., Huang B., Grumbach K. (2018). Maslach burnout inventory and a self-defined, single-item burnout measure produce different clinician and staff burnout estimates. J. Gen. Intern. Med..

[38] Lim W.Y., Ong J., Ong S., Hao Y., Abdullah H.R., Koh D.L., Mok U. (2019). The abbreviated maslach burnout inventory can overestimate burnout: A study of anesthesiology residents. J. Clin. Med..

[39] Montiel-Company J.M., Subirats-Roig C., Flores-Martí P., Bellot-Arcís C., Almerich-Silla J.M. (2016). Validation of the maslach burnout inventory-human services survey for estimating burnout in dental students. J. Dent. Educ..

[40] Talavera-Velasco B., Luceño-Moreno L., Martín-García J., García-Albuerne Y. (2018). Psychosocial risk factors, burnout and hardy personality as variables associated with mental health in police officers. Front. Psychol..

[41] Violanti J.M., Mnatsakanova A., Andrew M.E., Allison P., Gu J.K., Fekedulegn D. (2018). Effort–reward imbalance and overcommitment at work: Associations with police burnout. Police Q..

[42] Sánchez-Teruel D., Robles M.A. (2014). Personality and resilience in a special corps of the national police in Spain. J. Work Organ. Psychol..

